# Supplementary material for: The dual mechanism of social networks on the relationship between internationalization and firm performance: Empirical evidence from china
Source: PLoS One. 2022 Feb 11;17(2):e0263674. doi: 10.1371/journal.pone.0263674 (PMC8836315; doi:10.1371/journal.pone.0263674)
Supplement: Attachment — Submitted filename: Response to Reviewers.docx [file pone.0263674.s002.docx]

**PONE-D-21-26838(R1): Response to Reviewers**

Thank you very much for inviting me to submit this revision, and for your clear directions which I have used as main guidance. I appreciate your appraisal that my manuscript shows promise for making a valuable contribution. My point-by-point responses to your comments ( ***paraphrased in bolded italics***), are given below.

**RESPONSES TO THE EDITOR’S COMMENTS**

***Additional Editor Comments: Dear authors, I agree with the reviewer that you have to add more details in yuor methodology, better discuss the findings and better link your findings with prior theory. Also please engage in a better discussion on the research gaps you want to adress.***

Response: I have followed your suggestions, and do the following in the revised manuscript:

(1) I add more details in my methodology. I construct two econometric models, and I also introduce the methods of analyzing mediating effect and moderating effect.

(2) I further discuss the findings of the study. I emphasize that our research explains the reasons for the existence of dual mechanisms in social networks based on boundary spanning theory.

(3) In the introduction, I further discuss the relationship between current research and previous research. I explained that the existing research mainly focuses on the difference in intensity between business ties and political ties. However, the current research discusses the differences between the two from the perspective of impact mechanism.

**RESPONSES TO THE REVIEWER #1**

Thank you for your careful review of my manuscript, and for your positive assessment that this paper has a potential to make a nice contribution to the literature. Thank you also for your excellent suggestions on how I can improve the paper. I show below how and where I have responded to your comments. (I have paraphrased your comments in ***bolded italics***.)

***The research methodology should be improved The analysis needs to indicate to details and implications should be based on these results the introduction must indicate to main gaps and create a bridge between this part and literature review.***

Response: Thank you for these valid points. I have followed your suggestions, and do the following:

(1) In terms of research methods, I have constructed a mediating effect analysis model and a moderating effect analysis model to analyze the different roles of business ties and political ties.

(2) I introduced the details of the analysis method and explained the methods to test the mediating effect and moderating effect.

(3) I revised the introduction, briefly combed the research progress of the existing literature, and stressed that the existing research mainly focuses on the difference in strength between business ties and political ties. However, the current research analyzes the differences between the two from the perspective of impact mechanism, and explains the reasons for the differences based on boundary spanning theory.

(4) I further enrich the literature review of boundary spanning theory.

(5) I further discussed the results of the empirical study. In the discussion, I explained the support of the research results for the mediating effect of business ties and the moderating effect of political ties. Furthermore, I emphasize the role of boundary spanning theory in explaining the dual mechanism of social networks.

**Revision information of reference list**

In the revised manuscript, the references cited in the paper are also updated，the revised draft deleted two references and added 12 references.

In the old manuscript, two references were deleted as follows：

*12.	Iurkov, V.; Benito, G.R.G. Change in domestic network centrality, uncertainty, and the foreign divestment decisions of firms. J Int Bus Stud 2020, 51, 788-812.*

*13.	Cuypers, I.R.P.; Ertug, G.; Cantwell, J.; Zaheer, A.; Kilduff, M. Making connections: Social networks in international business. J Int Bus Stud 2020, 51, 714-736.*

In the revised manuscript, 12 references were added as follows：

*12.	Horak, S.; Taube, M. Same but different? Similarities and fundamental differences of informal social networks in China (guanxi) and Korea (yongo). Asia Pac J Manag 2016, 33, 595-616.*

*14.	Chen, C.C.; Chen, X.; Huang, S. Chinese guanxi: An integrative review and new directions for future research. Manage Organ Rev 2013, 9, 167-207.*

*15.	Xiao, Z.; Tsui, A.S. When brokers may not work: The cultural contingency of social capital in Chinese high-tech firms. Admin Sci Quart 2007, 52, 1-31.*

*16.	Wang, G.; Jiang, X.; Yuan, C.; Yi, Y. Managerial ties and firm performance in an emerging economy: Tests of the mediating and moderating effects. Asia Pac J Manag 2013, 30, 537-559.*

*17.	Arnoldi, J.; Villadsen, A.R. Political ties of listed Chinese companies, performance effects, and moderating institutional factors. Manage Organ Rev 2015, 11, 217-236.*

*20.	Zhang, M.; Qi, Y.; Wang, Z.; Zhao, X.; Pawar, K.S. Effects of business and political ties on product innovation performance: Evidence from China and India. Technovation 2019, 80, 30-39.*

*21.	Du, J.; Zhou, C. Does guanxi matter in the foreign expansion of Chinese manufacturing firms? The mediator role of linking and leveraging. Asia Pac J Manag 2019, 36, 473-497.*

*23.	Zhang, X.; Ma, X.; Wang, Y.; Li, X.; Huo, D. What drives the internationalization of Chinese SMEs? The joint effects of international entrepreneurship characteristics, network ties, and firm ownership. Int Bus Rev 2016, 25, 522-534.*

*24.	Li, J.; Xia, J.; Zajac, E.J. On the duality of political and economic stakeholder influence on firm innovation performance: T heory and evidence from C hinese firms. Strategic Manage J 2018, 39, 193-216.*

*25.	Shen, L.; Zhang, C.; Teng, W.; Du, N. How do business and political Networking shape overseas dispute resolution for state-owned enterprise from emerging economies. Int Bus Rev 2021, In Press.*

*36.	Anderson, J.C.; Narus, J.A. A model of distributor firm and manufacturer firm working partnerships. J Marketing 1990, 54, 42-58.*

*37.	Friedman, R.A.; Podolny, J. Differentiation of boundary spanning roles: Labor negotiations and implications for role conflict. Admin Sci Quart 1992, 28-47.*

So in sum, I feel that the above point-by-point responses can answer and address your concerns. Thank you again for all your suggestions. They have helped me greatly in strengthening this paper. Thank you very much and best wishes!
